# EP14 COVID-19 associated aortitis

**DOI:** 10.1093/rap/rkaa052.013

**Published:** 2020-11-03

**Authors:** Yun Zou, Bhavisha Vasta

**Affiliations:** Rheumatology Department, South Warwickshire NHS Foundation Trust, Warwick, United Kingdom

## Abstract

**Case report - Introduction:**

Since the emergence of Coronavirus disease 2019 (COVID-19) there has been increasing recognition of the potential associated cardio-vascular manifestations. There have been reports of Kawasaki like disease in children. However, in adults there are very few reports of non-cutaneous vasculitis. Here we report the case of an adult male presenting with an inflammatory aortitis associated with COVID-19 infection.

**Case report - Case description:**

A 71-year-old Caucasian male with a background of cholecystectomy and rotator cuff repair presented to hospital in May 2020 with a 3-month history of feeling generally unwell, weight loss and worsening thoraco-lumbar back pain. Prior to the onset of these symptoms he had had a 2-week illness in March 2020 clinically consistent with COVID-19 infection comprising fevers, hot sweats, dry cough, and chest tightness for which he had not sought medical attention. He had no recent travel history. Physical examination was unremarkable.

On admission, COVID-19 tests revealed evidence of prior infection with negative SARS-CoV-2 polymerase chain reaction test but positive SARS-CoV-2 antibodies. Blood tests revealed a marked inflammatory state with a C- reactive protein of 122mg/L, plasmas viscosity of 2.76, Ferritin 777ug/L, Interleukin-6 of 25 ng/L and normocytic anaemia with a Haemoglobin of 77g/L. Immunology tests were negative for anti-neutrophil cytoplasmic antibody, anti-glomerular basement antibodies, HLA-B27, anti-citrullinated protein antibody, rheumatoid factor, and nuclear antibodies, with normal IgG 4 subclasses. Microbiology workup showed negative blood cultures, syphilis screen and Hepatitis B and C serology. Temporal artery ultrasound was unremarkable. Troponin-T, pro-B-type natriuretic peptide, electrocardiogram and echocardiogram were normal. CT thorax abdomen pelvis revealed inflammatory change surrounding the aortic arch extending all the way down the aorta in keeping with a florid inflammatory aortitis with no aneurysms seen.

Rapid resolution of symptoms was seen with commencement of Prednisolone 40mg once daily, with normalisation of CRP one week later and subsequent normalisation of haemoglobin and plasma viscosity. A repeat CT aorta 2 weeks after commencement of prednisolone demonstrated a reduction in the thickness of the inflammatory rind over the aorta from 6mm to 2mm. The patient now continues a reducing regime of prednisolone and remains in clinical remission.

**Case report - Discussion:**

In children, Kawasaki like disease associated with COVID-19 is well described and can result in coronary artery inflammation and aneurysm. In adults, COVID-19 associated cutaneous vasculitis is well recognised however there are only a small number of case reports of organ specific vasculitis including the central nervous system, retina, and small bowel. To our knowledge this is the first reported case of aortitis associated with COVID-19 infection in an adult patient.

The mechanisms underlying the development of COVID-19 associated vasculitis are not established but may be secondary to endothelial inflammation. Findings from a histological case series suggest that SARS-CoV-2 can infect endothelial cells directly, possibly via endothelial ACE2 receptors, leading to inflammation in the endothelium. Another postulated mechanism is that endothelial cell dysfunction and inflammation is caused by the cytokine storm that can be seen in some patients with COVID-19 infection.

Our patient responded very well to corticosteroid treatment. However, in case of a relapse his cytokine profile could be helpful in directing further therapeutic options. IL-6 levels were elevated in our patient. Studies show that IL-6 appears to play a dominant role in the cytokine storm. In a report of 150 patients IL-6 was found to be significantly higher in the group with severe disease and possibly predictive of mortality. The IL-6 antagonist, Tocilizumab, has also been used with promising results. The first report of its use was in China in 21 critically ill COVID-19 patients with significant improvements. Since this first report, further clinical trials are underway investigating the efficacy and tolerability of IL-6 antagonists in patients with COVID-19 disease. Expanding our understanding of the pathogenesis of COVID-19 associated vasculitis is a critical area for future research to identify other immune targets for novel/ existing therapeutic agents.

**Case report - Key learning points**
 

1. Vasculitis including aortitis can be a complication of COVID-19 infection.
2. Endothelial cell inflammation is likely to play key role in the pathogenesis of COVID-19 associated vasculitis.
3. In addition to corticosteroids, other immune-modulating drugs presently used in rheumatology may be effective therapeutic agents.

